# How do Design Characteristics Affect Respondent Engagement? Assessing Attribute Non-attendance in Discrete Choice Experiments Valuing the EQ-5D-5L

**DOI:** 10.1007/s40271-025-00735-9

**Published:** 2025-03-15

**Authors:** Peiwen Jiang, Deborah Street, Richard Norman, Rosalie Viney, Mark Oppe, Brendan Mulhern

**Affiliations:** 1 Faculty of Health, Centre for Health Economics Research and Evaluation, University of Technology Sydney, Broadway, PO Box 123, Sydney, NSW 2007 Australia; 2 Population Health Research & Epidemiology, South Western Sydney Local Health District, Sydney, NSW Australia; 3 School of Population Health, Curtin University, Perth, WA Australia; 4 Section Medical Psychology and Psychotherapy, Department of Psychiatry, Erasmus MC, Erasmus University, Rotterdam, The Netherlands

## Abstract

**Introduction:**

Discrete choice experiments (DCEs) are increasingly applied to develop value sets for health-related quality-of-life instruments, but respondents may adopt various simplifying heuristics that affect the resulting health state values. Attribute level overlap can make these DCE tasks easier and thereby increase respondent engagement. This study uses choice tasks involving EQ-5D-5L health states to compare designs with and without overlap, constructed using different methods (generator-developed design, Ngene, SAS, and Bayesian D-efficient design) to assess respondent non-attendance to attributes.

**Methods:**

A multi-arm DCE using the EQ-5D-5L was conducted in the Australian general population. The performance of designs with various properties was compared using the level of respondent engagement. Respondent engagement was quantified through the inferred attribute non-attendance (ANA) estimated by the equality constrained latent class model. Utility decrements derived using all respondents (i.e., including non-attendees) were compared with estimates obtained only from those who attended to all EQ-5D-5L attributes.

**Results:**

The inclusion of overlap improved full attendance rates from 22.3–28.4% to 28.2–54.2%. Within designs with overlap, modified Fedorov designs (constructed using either Ngene or SAS macros) had higher full attendance rates than other designs. The relative attribute importance of the EQ-5D-5L also differed significantly before and after data exclusion using ANA analysis, but there was no clear pattern in the differences.

**Conclusions:**

This study found evidence to support the use of modified Fedorov designs (constructed using Ngene or SAS) with attribute overlap to reduce ANA and improve respondent engagement in DCE studies. It highlights the potential value of ANA analysis as a quality-control tool for the inclusion and exclusion of respondents in future health valuation work for the EQ-5D-5L.

**Supplementary Information:**

The online version contains supplementary material available at 10.1007/s40271-025-00735-9.

## Key Points for Decision Makers

**Table:** 

Modified Fedorov designs implemented in Ngene or SAS with attribute overlap are recommended to reduce attribute non-attendance (ANA) and enhance respondent engagement in discrete choice experiments (DCEs).
ANA models can be used to exclude respondents who did not attend to any attributes and to identify partial attenders for further robustness checks, improving the quality of the data for analysis.
Future research should focus on developing advanced models capable of disentangling the effects of preference heterogeneity and attribute attendance.

## Introduction

Attribute non-attendance (ANA) is a phenomenon in discrete choice experiments (DCEs) whereby respondents consider only one or a few attributes presented in a choice task [1]. ANA can arise for two main reasons. First, it may reflect genuine preferences: some respondents may ignore certain attributes simply because they do not consider them relevant to their decision-making. In this case, ANA is an indication that those attributes are not important and do not meaningfully influence their choices. Second, ANA can occur as a decision heuristic, where respondents actively ignore attributes, not because they are unimportant but as a strategy to simplify complex tasks.

Heuristic-driven ANA is particularly likely to occur in highly complex choice tasks where respondents are required to consider multiple attributes presented at different levels at the same time. Design construction methods in DCEs are specifically developed to optimise the statistical efficiency of the experiment, aiming to maximise the information obtained from respondents’ choices while minimising the required sample size. However, there is a trade-off between behavioural efficiency and statistical efficiency.

Despite this need for balance, most studies evaluating DCE design methods have focused on statistical efficiency alone, with limited attention to the behavioural implications of different construction techniques [2–4]. The choice of construction method—such as orthogonal designs (which maintain attribute level independence) or efficient designs (which relax orthogonality constraints to optimise information yield)—can influence the rate of ANA [1, 5]. A study focusing on preferences of healthcare providers compared ANA between orthogonal and efficient designs using the equality constrained latent class (ECLC) model [5]. They found higher levels of ANA in efficient designs than in orthogonal designs and a more pronounced difference among illiterate respondents than among literate respondents. Another study on threatened species in New Zealand also adopted ECLC models to evaluate behavioural efficiency between orthogonal, generator-developed, and efficient designs [6]. Their results showed that the orthogonal design resulted in the lowest rate of full attendance and the D-efficient design achieved the highest level of full attendance. These contrasting findings suggest that construction methods may affect ANA differently.

In light of the increasing importance placed on respondent efficiency, attribute overlap has been recognised as a useful strategy to improve attribute attendance [7, 8]. Various design methods have included this feature in the construction of DCE designs [9–11]. Attribute overlap refers to the practice of setting a subset of attributes at the same level across options within the choice task. Jonker et al. [8] employed a Bayesian D-efficiency design to compare designs with no overlapping attributes and those with three of six attributes overlapped, using the EQ-5D-5L as the descriptive system. Their study showed that designs with attribute overlap significantly improved attribute attendance, increasing the number of attended attributes from two to three of a total of five.

Both studies comparing DCE construction methods—one focused on healthcare provider preferences and the other on threatened species—used designs without attribute overlap [5, 6]. Despite this similarity, their findings differed significantly, suggesting that construction method alone may not fully explain variations in ANA. Attribute overlap has been proposed as a potential way to enhance both respondent efficiency and attribute attendance, potentially playing a more influential role than construction method alone. This highlights the need to examine both construction method and attribute overlap together to understand their combined effects on respondent behaviour.

The growing interest in integrating attribute overlap in DCEs has led to its incorporation into various DCE construction algorithms, such as Ngene [9], SAS macros [10], and generator-developed designs [11]. It is surprising that no investigation has yet explored the ANA among designs featuring level overlap, particularly those constructed via differing methods. Furthermore, there remains a notable gap in the realm of preference measures, with limited exploration of design methodologies and structures, except for the studies by Jonker et al. [8, 12]. The current study aims to fill this knowledge gap and contribute to the literature by comprehensively comparing various design construction methods while considering the impact of attribute overlap in the domain of preference measures.

## Methods

### Choice Experiment

**Fig. 1:**
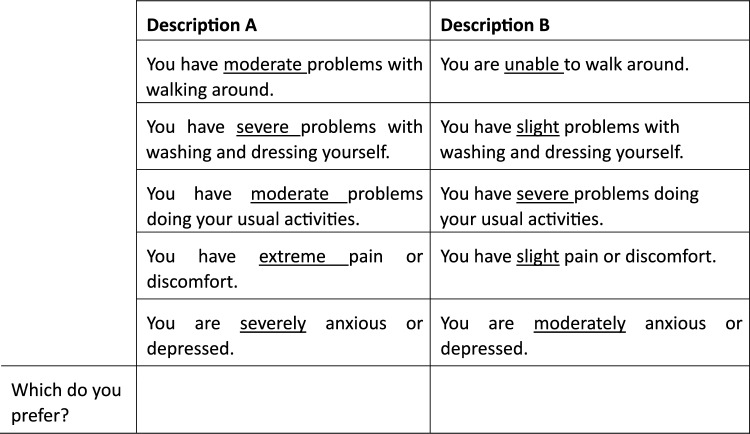
Context and typical choice set. Please consider, and imagine living with, one of the two health states described below. Then tell us which description you would prefer to experience.

The descriptive system used in this study is the most widely used health-related quality-of-life instrument, the EQ-5D-5L. It contains five attributes—mobility, self-care, usual activities, pain/discomfort, and anxiety/depression—each presented at one of five levels (no problems, slight problems, moderate problems, severe problems, and extreme problems/ unable to). Health states are described by the quintuples of levels, with 11111 denoting the best health state possible and 55555 representing the worst health state possible. There were two hypothetical situations within each choice task, and respondents were asked to choose which they preferred, as shown in Fig. 1.

The study included a total of 19 designs constructed using six construction methods (Generator-developed design, modified Fedorov algorithm implemented in Ngene, modified Fedorov algorithm implemented in SAS, Bayesian D-efficient design, STATA, and modified coordinate–exchange algorithm implemented in Ngene), using zero and non-zero priors, and including attribute level overlap on two of the five attributes or on none (see Mulhern [13] for details). Each respondent was randomly allocated to one of the 19 designs, and a total of 21 choice sets were presented to these respondents. In this article, we focus on a subset of four construction methods capable of generating designs with attribute overlap and only on designs constructed using zero priors. We selected only these designs to ensure comparability because efficient designs can be affected significantly by misspecifications in the priors [3]. By exclusively considering designs with zero priors, we effectively eliminated bias and enhanced the validity of our comparisons.

The four construction methods included in this article are generator-developed design [11], Ngene [9], SAS macros [10], and Bayesian D-efficient design algorithm implemented in R by Oppe and van Hout [14] based on Rose et al. [15]. Each of these construction methods was used to generate one design with no overlap and another with two overlapping attributes out of five. This resulted in a total of eight designs, which are briefly outlined below.

The generator-developed design started with an initial set of profiles that form an orthogonal array for five attributes each with five levels, and a set of generators [11]. To obtain a 100% efficient design for zero priors for a design with no overlapping attributes, two generators are required. For each attribute, the two generators must between them have one entry that is 1 or 4 and one entry that is 2 or 3. In addition, our selection of generators was strategically aimed at minimising the number of dominant pairs within the design. Therefore, for the design with no overlapping attributes, the generators were (1,1,1,2,2) and (2,2,2,4,4). For the design with two overlapping attributes, we elected to again have two generators. To obtain attributes with equal levels in two of the attributes in the two options, a generator must have two elements that are equal to 0. We used the generators (1,1,1,0,0) and (0,0,2,1,1).

The modified Fedorov algorithm was used in both the Ngene software and the SAS macros to compare software implementations rather than to focus on different design algorithms. For designs with no overlap, the algorithm iteratively exchanges a profile in the design with one from all possible candidate profiles until no substantial improvement in the D-error value is observed. With regards to designs with overlap, a candidate set of choice sets consisting of all 40,000 pairs with two overlapping attributes was used in both Ngene and SAS. These choice sets then underwent iteration to identify the optimal design with overlap on two attributes.

The fourth construction method we included was the Bayesian D-efficient design algorithm implemented in R by Oppe and van Hout [14] based on Rose et al. [15]. Overlapping and non-overlapping designs were generated, subject to the constraints that no pair could be duplicated, choice sets could not include dominated options, and the designs needed to meet a specific requirement for level balance (see the electronic supplementary material [ESM]-A).

The survey consisted of several sections. It began with basic demographic questions (age, gender, and region) to ensure quota distribution, followed by survey details and consent. Participants then completed a self-reported EQ-5D-5L assessment before receiving instructions on the DCE tasks. This was followed by 21 DCE choice tasks. Finally, respondents answered follow-up questions regarding survey difficulty and additional demographic details.

### Sample Recruitment

A total of 3365 respondents were recruited by an Australian online panel company, Pureprofile. The panel company gave a small incentive to respondents who fully completed the survey. The study sample was representative of the Australian general population in terms of age, gender, and region. As mentioned, 8 of 19 designs were selected for this study, so we used a subset of the sample (*N* = 1432). The quota allocation (age, gender, and region) was applied to each design to minimise the potential impact of these demographics on the results.

### Attribute Non-attendance

The performance of the designs was compared by evaluating ANA. The ECLC model was used to identify the variations in ANA patterns to the dimensions of the EQ-5D-5L across these designs. Unlike the commonly used latent class model that focuses on preference heterogeneity, the ECLC model defines classes based on respondents’ behaviour regarding attribute attendance. A high membership probability in a class indicates a greater likelihood of engaging in the attribute attendance behaviour associated with that class. This model not only generates preference estimates but also provides information on the probability of each possible ANA strategy for each individual. When an attribute is ignored, its coefficient is assumed to be zero. For attributes that are considered, they are set to have the same values across all classes, assuming homogeneous taste parameters. However, information-processing behaviour is assumed to be heterogeneous.

There are 32 (= 2^5^) possible ANA strategies for the choice sets used, ranging from attending to each dimension to ignoring all dimensions of the EQ-5D-5L and choosing the preferred health state randomly. Other possible ANA strategies include nonattendance to one, two, three, or four of the five attributes. These strategies translate into 32 possible classes within the ECLC model. However, including all 32 classes simultaneously could result in issues of over-identification. To address this, a five-step approach was adopted to include and exclude ANA patterns. Classes with a membership probability below 5% were deemed insignificant and therefore excluded from the model, as suggested by Doherty et al. [16]. Only significant classes were retained in the ECLC model. The estimation of the ECLC model began with six classes, including one class representing full attendance and five classes featuring non-attendance to exactly one of the five dimensions. In the second step, nonsignificant classes from step one were removed and the next 10 = ${C}_{2}^{5}$ processing strategies where respondents ignored exactly two attributes were included in the model estimation. Similarly, the model in step three removed nonsignificant classes from step two and included the next 10 = ${C}_{3}^{5}$ classes representing respondents assumed to have ignored exactly three attributes. In step four, the model was re-estimated including significant classes from step three, along with five ANA patterns = ${C}_{4}^{5}$ where respondents considered only one dimension, and a class where all attributes were ignored. Finally, the resulting ECLC model, consisting only of significant classes, was used to generate estimates for the EQ-5D-5L.

### Statistical Modelling

The multinomial logit (MNL) model was employed to analyse the choice data. The utility for respondent *i* associated with option *j* in choice task *t* is given by

1 $$U_{ijt} = x_{ijt} \beta + \epsilon_{ijt} .$$

where *x*_*ijt*_ is a vector of dummy coded attribute levels shown to individual* i* as option *j* in choice task *t*, and *β* is a preference vector for the effects of these attribute levels, assuming preference homogeneity. The error term $\epsilon_{ijt}$ is assumed to have a standard type I extreme value distribution. The probability of respondent *i* choosing option *j* in choice task* t* under the MNL framework can be described as:

2 $$P_{ijt} \vee \beta = \frac{{e^{{\beta x_{ijt} }} }}{{\mathop \sum \nolimits_{j = 1}^{J} e^{{\beta x_{ijt} }} }}.$$

The MNL model assigns a weight to each dimension for every respondent, even if respondents might not engage with the dimensions. To address this issue, the ECLC model was used to explore different ANA patterns. In this model, individuals are classified into latent classes based on their ANA patterns, and the parameter of class *c* is denoted by *β*_*c*_. Across all classes, preference parameters are assumed to be the same except for the non-attended dimensions, and hence are the same as the MNL model. For non-attended dimensions, their parameters are set to be zero. The probability of observing the ANA pattern *c* for individual *i* choosing option* j* in choice task *t* can be written as:

3 $$P_{ijt} \vee \beta_{c} = \frac{{e^{{\beta_{c} x_{ijt} }} }}{{\mathop \sum \nolimits_{j = 1}^{J} e^{{\beta_{c} x_{ijt} }} }}.$$

Based on the ANA analysis, respondents who have attended all five attributes (full attenders) and those who did not attend all five (partial attenders) can be identified. To test for differences in estimates by full attendance status, the data were first analysed using the MNL model with main effects and interactions between a dummy indicating full attendance or not, and each attribute level.

In addition, separate MNL main effects models were estimated for all respondents and full attenders. This was conducted to compare the EQ-5D-5L coefficients before and after exclusion, employing ANA analysis as a quality check procedure. To compare MNL results across different models, the coefficients were anchored using the coefficient for the worst EQ-5D-5L state (55555). Furthermore, we examined the relative attribute importance (RAI) scores both before and after data exclusion. The RAI scores were calculated by dividing the sum of all coefficients for each dimension by the sum of all coefficients, as shown in Eq. 4.

4 $$RAI_{k} = \frac{{\beta_{k} }}{{\beta_{MO} + \beta_{SC} + \beta_{UA} + \beta_{PD} + \beta_{AD} }}$$

where ${\beta }_{k}$ is the sum of all coefficients for each dimension of the EQ-5D-5L.

An external validity analysis was conducted to assess the extent to which the ECLC model’s attendance classifications align with respondents’ levels of engagement and attentiveness. Specifically, the presence of straightlining (i.e., selecting the same option repeatedly, e.g., AAAA … or BBBB …), completion times for both the choice tasks and the overall survey, and feedback question responses regarding task difficulty and consideration of the entire description were examined. These indicators were then compared across the three attendance classes (full attenders, partial attenders, and non-attenders) to determine whether the observed behaviours matched the expected pattern of decreasing attentiveness and engagement.

The ANA analysis was performed in Latent GOLD 5.1 [17], and the MNL models were estimated in R [18] and RStudio [19], using the *gmnl* package [20].

## Results

### Respondent Characteristics

The characteristics of the overall sample are similar to those of the overall Australian population in terms of age, gender, and region at the state and territory level. There is no significant difference between the subsamples at the 0.05 level by age, gender, or region for the overlap or non-overlap groups. This is also the case for most of the other demographic characteristics measured. However, the respondents are more highly educated than the overall Australian population. At the overall level, 47% of the sample reported having a long-term health condition, and 22% reported themselves to be in the best EQ-5D-5L health state.

### ANA Probabilities

Table 1 presents estimated probabilities from the ECLC model. For designs without overlap, the generator-developed designs showed the highest probability of attending to all five EQ-5D attributes (28.4%), whereas the lowest estimated full attendance rate was observed in SAS designs (22.3%). The lowest probability of ignoring all attributes was found in Ngene designs (8.5%) and the highest in R designs (17.8%).

**Table 1:** Estimated class probabilities from the final equality constrained latent class (ECLC) model for each design

No overlap				Overlap			
Class	Attributes non-attended	Prob. (%)	95% CI	Class	Attributes non-attended	Prob. (%)	95% CI
Gen-dev							
**1**	**None**	**28.4**	**28.3–28.6**	**1**	**None**	**30.8**	**30.7–31.0**
2	PD+AD	6.3	6.2–6.3	2	AD	21.2	21.1–21.4
3	MO+UA+AD	5.1	5.0–5.1	3	MO+SC	9.9	9.8–10.0
4	MO+SC+UA+PD	15.7	15.6–15.7	4	PD+AD	16.6	16.4–16.7
5	MO+SC+UA+AD	9.3	9.2–9.4	**5**	**MO+SC+UA+PD+AD**	**21.5**	**21.4–21.6**
6	MO+UA+PD+AD	8.2	8.1–8.2				
7	SC+UA+PD+AD	12.1	12.0–12.1				
**8**	**MO+SC+UA+PD+AD**	**15.0**	**15.0–15.2**				
Ngene							
**1**	**None**	**24.6**	**24.6–24.9**	**1**	**None**	**54.2**	**54.2–54.4**
2	AD	9.7	9.5–9.7	2	PD+AD	22.9	22.8–23.1
3	MO+SC	12.5	12.4–12.6	**3**	**MO+SC+UA+PD+AD**	**22.9**	**22.7–22.9**
4	SC+PD+AD	8.5	8.4–8.5				
5	UA+PD+AD	8.6	8.5–8.7				
6	MO+SC+UA+PD	14.3	14.3–14.4				
7	MO+UA+PD+AD	8.9	8.8–8.9				
8	SC+UA+PD+AD	4.3	4.3–4.4				
**9**	**MO+SC+UA+PD+AD**	**8.5**	**8.5–8.6**				
SAS							
**1**	**None**	**22.3**	22.1–22.3	**1**	**None**	**49.4**	**49.4–49.8**
2	SC	9.8	9.7–9.9	2	SC+UA	14.3	14.1–14.3
3	MO+SC+UA	5.8	5.6–5.8	3	UA+AD	10.4	10.3–10.4
4	UA+PD+AD	16.8	16.7–16.9	4	PD+AD	8.2	8.1–8.3
5	MO+SC+UA+PD	12.9	12.9–13.0	**5**	**MO+SC+UA+PD+AD**	**17.6**	**17.5–17.7**
6	MO+SC+UA+AD	5.6	5.5–5.7				
7	SC+UA+PD+AD	14.6	14.5–14.7				
**8**	**MO+SC+UA+PD+AD**	**12.3**	**12.1–12.3**				
R							
**1**	**None**	**22.6**	**22.4–22.7**	**1**	**None**	**28.2**	**28.1–28.3**
2	MO	9.8	9.6–9.8	2	AD	8.7	8.6–8.8
3	AD	21.6	21.6–21.8	3	MO+SC	16.1	16.0–16.2
4	MO+SC+UA	6.1	6.0–6.2	4	PD+AD	15.4	15.3–15.5
5	MO+SC+UA+PD	9.4	9.3–9.4	5	SC+UA+PD	9.2	9.1–9.2
6	SC+UA+PD+AD	12.8	12.8–12.9	6	SC+UA+PD+AD	8.4	8.2–8.4
**7**	**MO+SC+UA+PD+AD**	**17.8**	**17.6–17.8**	**7**	**MO+SC+UA+PD+AD**	**14.1**	**14.0–14.2**

Bold text indicates full attendance and non-attendance classes

*AD* anxiety/depression, *CI* confidence interval, *Gen-dev* generator-developed designs [11], *MO* mobility, *Ngene* modified Fedorov designs constructed in Ngene [9], *PD* pain/discomfort, *Prob.* probability, *R* Bayesian D-efficient design algorithm implemented in R by Oppe and van Hout [14] based on Rose et al. [15], *SAS* modified Fedorov designs constructed in SAS [10], *SC* self-care, *UA* usual activities

More importantly, the inclusion of attribute overlap had a significant impact on the inferred attendance rates for all attributes, regardless of the specific design method employed. It is worth noting that Ngene and SAS designs had the most significant improvements. The attendance estimates for these two methods without attribute overlap were at 24.6% and 22.3%, respectively. When examining designs with attribute overlap, these rates increased to 54.2% and 49.4%, respectively. An intriguing observation is that attribute overlap not only increased the estimated rate of attendance to all attributes in the generator-developed, Ngene, and SAS designs but also increased the estimated rate of non-attendance (i.e., none of the dimensions being considered). However, this effect was not observed in the R designs.

### Health State Valuation

Table 2 focuses on designs with overlap and presents estimates derived from MNLs with main effects and interactions between a dummy indicating whether or not the respondent considered all attribute levels (i.e., reflecting partial attenders and full attenders). The corresponding estimates derived from designs with no overlap can be found in Table 3. Most of the main effects of the dimensions were statistically different from zero. Several interaction terms were statistically significant, especially for levels 4 and 5 in each attribute. This suggests that attribute attendance influenced the decrements of these attribute levels. The number of statistically significant interaction terms ranged from 13 to 17 in designs with overlap and from 12 to 15 in designs without overlap. Furthermore, most significant parameter magnitudes were larger among full attenders than among partial attenders. For example, examining MO3 for the generator-developed design with overlap in Table 2, the decrements of MO3 for partial attenders and full attenders were significantly different, at − 0.427 and − 1.168 (− 0.427 to − 0.741), respectively. However, there was one exception: the magnitude of level 2 in the mobility dimension in the R design with overlap was larger in partial attenders than in full attenders.

**Table 2:** Estimates from multinomial logit models with full attendance interactions for designs with overlap

	Gendev			Ngene			SAS			R		
	Coef	SE	*P*	Coef	SE	*P*	Coef	SE	*P*	Coef	SE	*P*
MO2	− 0.216	0.121	0.074	− 0.246	0.139	0.077	− 0.236	0.139	0.089	− 0.414	0.121	0.001
MO3	− 0.427	0.151	0.005	− 0.234	0.151	0.122	− 0.601	0.147	< 0.001	− 0.277	0.116	0.017
MO4	− 0.937	0.156	< 0.001	− 0.665	0.168	< 0.001	− 0.672	0.140	< 0.001	− 0.800	0.129	< 0.001
MO5	− 1.197	0.132	< 0.001	− 1.148	0.166	< 0.001	− 1.536	0.156	< 0.001	− 1.025	0.130	< 0.001
SC2	0.027	0.123	0.824	0.036	0.141	0.800	− 0.198	0.149	0.183	0.087	0.129	0.498
SC3	− 0.322	0.152	0.034	0.059	0.154	0.703	− 0.169	0.146	0.247	0.014	0.147	0.923
SC4	− 0.826	0.154	< 0.001	− 0.457	0.150	0.002	− 0.575	0.151	< 0.001	− 0.325	0.150	0.030
SC5	− 1.160	0.131	< 0.001	− 0.591	0.137	< 0.001	− 0.723	0.146	< 0.001	− 0.596	0.144	< 0.001
UA2	− 0.236	0.088	0.007	0.226	0.136	0.095	0.211	0.149	0.158	− 0.139	0.137	0.312
UA3	− 0.483	0.087	< 0.001	0.083	0.146	0.569	0.048	0.140	0.731	− 0.102	0.136	0.453
UA4	− 1.052	0.091	< 0.001	− 0.092	0.134	0.490	0.098	0.147	0.502	− 0.218	0.140	0.119
UA5	− 1.354	0.094	< 0.001	− 0.545	0.140	< 0.001	− 0.329	0.139	0.018	− 0.486	0.174	0.005
PD2	− 0.199	0.123	0.106	0.049	0.156	0.753	0.114	0.136	0.404	− 0.118	0.129	0.360
PD3	− 0.230	0.154	0.135	0.147	0.142	0.304	− 0.389	0.136	0.004	− 0.255	0.133	0.055
PD4	− 0.829	0.158	< 0.001	− 0.037	0.154	0.808	− 0.691	0.131	< 0.001	− 0.661	0.115	< 0.001
PD5	− 1.043	0.135	< 0.001	− 0.096	0.128	0.454	− 0.856	0.140	< 0.001	− 0.601	0.146	< 0.001
AD2	− 0.038	0.130	0.770	0.139	0.182	0.446	0.364	0.134	0.006	0.219	0.168	0.194
AD3	− 0.128	0.155	0.407	− 0.277	0.190	0.144	− 0.071	0.134	0.595	− 0.171	0.143	0.229
AD4	− 0.341	0.157	0.030	0.407	0.185	0.028	− 0.407	0.132	0.002	− 0.818	0.147	< 0.001
AD5	− 0.439	0.135	0.001	0.050	0.148	0.738	− 0.633	0.135	< 0.001	− 0.863	0.132	< 0.001
Interaction with full attendance												
MO2	− 0.111	0.264	0.673	**− 0.603**	0.209	0.004	**− 0.443**	0.224	0.048	0.070	0.281	0.802
MO3	**− 0.741**	0.335	0.027	**− 0.653**	0.231	0.005	**− 0.777**	0.268	0.004	− 0.344	0.290	0.236
MO4	**− 1.121**	0.350	0.001	**− 1.221**	0.258	< 0.001	**− 2.013**	0.277	< 0.001	**− 0.817**	0.289	0.005
MO5	**− 1.904**	0.316	< 0.001	**− 1.421**	0.266	< 0.001	**− 1.513**	0.311	< 0.001	**− 1.275**	0.318	< 0.001
SC2	− 0.309	0.274	0.258	− 0.146	0.225	0.515	0.002	0.272	0.993	**− 1.378**	0.318	< 0.001
SC3	**− 0.841**	0.333	0.012	**− 0.512**	0.249	0.040	**− 0.477**	0.237	0.044	**− 0.979**	0.336	0.004
SC4	**− 1.315**	0.343	< 0.001	**− 0.661**	0.245	0.007	**− 1.652**	0.277	< 0.001	**− 1.905**	0.397	< 0.001
SC5	**− 1.936**	0.313	< 0.001	**− 1.072**	0.249	< 0.001	**− 2.293**	0.299	< 0.001	**− 2.803**	0.452	< 0.001
UA2	− 0.284	0.200	0.157	− 0.256	0.202	0.206	0.084	0.289	0.771	− 0.359	0.305	0.239
UA3	− 0.235	0.210	0.263	**− 0.995**	0.229	< 0.001	− 0.341	0.266	0.200	− 0.358	0.301	0.234
UA4	**− 0.903**	0.248	< 0.001	**− 1.044**	0.216	< 0.001	**− 1.874**	0.252	< 0.001	**− 1.518**	0.352	< 0.001
UA5	**− 0.879**	0.225	< 0.001	**− 0.860**	0.213	< 0.001	**− 2.321**	0.256	< 0.001	**− 1.590**	0.412	< 0.001
PD2	− 0.328	0.279	0.241	**− 0.558**	0.242	0.021	− 0.280	0.235	0.234	0.032	0.323	0.921
PD3	− 0.518	0.356	0.145	**− 0.959**	0.226	< 0.001	**− 0.740**	0.242	0.002	**− 0.809**	0.302	0.007
PD4	**− 1.782**	0.407	< 0.001	**− 2.072**	0.256	< 0.001	**− 1.796**	0.256	< 0.001	**− 2.502**	0.323	< 0.001
PD5	**− 2.403**	0.358	< 0.001	**− 2.667**	0.231	< 0.001	**− 1.864**	0.286	< 0.001	**− 2.971**	0.433	< 0.001
AD2	− 0.587	0.330	0.075	− 0.242	0.295	0.412	**− 1.027**	0.250	< 0.001	− 0.667	0.419	0.112
AD3	**− 1.640**	0.390	< 0.001	**− 0.747**	0.276	0.007	**− 1.103**	0.240	< 0.001	**− 1.296**	0.349	< 0.001
AD4	**− 3.253**	0.405	< 0.001	**− 2.581**	0.289	< 0.001	**− 2.262**	0.289	< 0.001	**− 3.464**	0.493	< 0.001
AD5	**− 4.247**	0.429	< 0.001	**− 2.610**	0.245	< 0.001	**− 2.150**	0.319	< 0.001	**− 2.950**	0.439	< 0.001
Significant interaction terms (*n*)			13			17			16			14
AIC			3941			3666			3452			4164
BIC			4189			3913			3700			4411
L L			− 1930			− 1793			− 1686			− 2042

*AD* anxiety/depression, *AIC* Akaike information criterion, *BIC* Bayesian information criterion, *Coef* coefficient estimate, *Gen-dev* generator-developed designs [11], *LL* log-likelihood, *MO* mobility, *Ngene* modified Fedorov designs constructed in Ngene [9], *Obs* number of observations, *P* p-value, *PD* pain/discomfort, *R* Bayesian D-efficient design algorithm implemented in R by Oppe and van Hout [14] based on Rose et al. [15], *SAS* modified Fedorov designs constructed in SAS [10], *SC* self-care, *SE* standard error, *UA* usual activities

Significant interaction terms are in bold

**Table 3:** Estimates from multinomial logit models with full attendance interactions for designs with no overlap

	Gendev			Ngene			SAS			R		
	Coef	SE	*P*	Coef	SE	*P*	Coef	SE	*P*	Coef	SE	*P*
MO2	− 0.272	0.085	0.001	− 0.083	0.085	0.329	− 0.177	0.085	0.036	0.031	0.116	0.791
MO3	− 0.214	0.087	0.014	− 0.157	0.088	0.075	− 0.276	0.087	0.001	− 0.038	0.096	0.690
MO4	− 0.599	0.087	< 0.001	− 0.723	0.093	< 0.001	− 0.811	0.090	< 0.001	− 0.447	0.108	< 0.001
MO5	− 0.853	0.089	< 0.001	− 0.989	0.100	< 0.001	− 1.099	0.090	< 0.001	− 0.663	0.109	< 0.001
SC2	− 0.151	0.084	0.074	− 0.245	0.085	0.004	− 0.110	0.086	0.200	− 0.007	0.104	0.945
SC3	− 0.191	0.087	0.027	− 0.143	0.088	0.104	− 0.104	0.086	0.224	0.018	0.101	0.860
SC4	− 0.429	0.087	< 0.001	− 0.681	0.092	< 0.001	− 0.397	0.087	< 0.001	− 0.364	0.100	< 0.001
SC5	− 0.597	0.090	< 0.001	− 0.687	0.093	< 0.001	− 0.658	0.087	< 0.001	− 0.680	0.118	< 0.001
UA2	− 0.129	0.085	0.128	0.055	0.091	0.545	− 0.110	0.085	0.198	− 0.152	0.111	0.170
UA3	− 0.210	0.087	0.016	− 0.061	0.095	0.518	− 0.136	0.086	0.115	− 0.058	0.111	0.601
UA4	− 0.281	0.087	0.001	− 0.238	0.087	0.006	− 0.199	0.087	0.022	− 0.418	0.099	< 0.001
UA5	− 0.384	0.089	< 0.001	− 0.399	0.085	< 0.001	− 0.267	0.090	0.003	− 0.640	0.108	< 0.001
PD2	− 0.143	0.083	0.084	− 0.131	0.092	0.155	− 0.094	0.086	0.276	0.110	0.112	0.328
PD3	− 0.295	0.085	0.001	− 0.275	0.092	0.003	− 0.124	0.086	0.151	0.069	0.127	0.587
PD4	− 0.493	0.083	< 0.001	− 0.517	0.092	< 0.001	− 0.485	0.082	< 0.001	− 0.375	0.092	< 0.001
PD5	− 0.706	0.087	< 0.001	− 0.694	0.095	< 0.001	− 0.602	0.085	< 0.001	− 0.543	0.119	< 0.001
AD2	0.047	0.083	0.570	− 0.121	0.086	0.160	− 0.306	0.084	< 0.001	0.101	0.106	0.342
AD3	− 0.137	0.083	0.100	− 0.081	0.090	0.369	− 0.374	0.086	< 0.001	− 0.182	0.124	0.142
AD4	− 0.582	0.083	< 0.001	− 0.733	0.095	< 0.001	− 0.793	0.090	< 0.001	− 0.615	0.117	< 0.001
AD5	− 0.573	0.087	< 0.001	− 0.830	0.090	< 0.001	− 0.845	0.089	< 0.001	− 0.773	0.109	< 0.001
Interaction with full attendance												
MO2	− 0.207	0.227	0.360	0.083	0.234	0.724	− 0.362	0.262	0.167	− 0.153	0.384	0.690
MO3	**− 0.706**	0.246	0.004	**− 0.709**	0.244	0.004	− 0.087	0.298	0.771	**− 0.885**	0.260	0.001
MO4	**−1.369**	0.276	< 0.001	**− 0.898**	0.255	< 0.001	**− 0.882**	0.328	0.007	**−1.526**	0.381	< 0.001
MO5	**−1.995**	0.273	< 0.001	**−1.517**	0.290	< 0.001	**−1.324**	0.325	< 0.001	**−1.788**	0.367	< 0.001
SC2	− 0.292	0.221	0.185	− 0.382	0.230	0.097	− 0.399	0.282	0.156	**− 0.785**	0.310	0.011
SC3	− 0.169	0.253	0.506	**− 0.973**	0.226	< 0.001	− 0.452	0.284	0.111	**−1.257**	0.336	< 0.001
SC4	**−1.241**	0.259	< 0.001	**−1.479**	0.259	< 0.001	**−1.948**	0.269	< 0.001	**−1.967**	0.358	< 0.001
SC5	**−1.743**	0.291	< 0.001	**−1.423**	0.271	< 0.001	**−1.977**	0.305	< 0.001	**−2.178**	0.385	< 0.001
UA2	− 0.105	0.219	0.631	0.071	0.241	0.768	− 0.439	0.273	0.109	− 0.078	0.324	0.809
UA3	− 0.217	0.262	0.408	− 0.181	0.261	0.488	**− 0.603**	0.272	0.027	− 0.321	0.313	0.305
UA4	**− 0.545**	0.266	0.040	**− 0.930**	0.234	< 0.001	**−1.653**	0.341	< 0.001	**−1.354**	0.304	< 0.001
UA5	**−1.344**	0.265	< 0.001	**−1.112**	0.227	< 0.001	**− 2.191**	0.363	< 0.001	**− 1.562**	0.306	< 0.001
PD2	0.100	0.212	0.637	− 0.324	0.234	0.165	− 0.129	0.266	0.629	− 0.259	0.351	0.461
PD3	− 0.073	0.217	0.736	**− 0.641**	0.236	0.007	− 0.254	0.283	0.369	− 0.611	0.368	0.097
PD4	**− 1.065**	0.214	< 0.001	**− 1.700**	0.263	< 0.001	**− 1.297**	0.288	< 0.001	**− 1.485**	0.329	< 0.001
PD5	**− 1.704**	0.257	< 0.001	**− 1.718**	0.278	< 0.001	**− 1.903**	0.404	< 0.001	**− 1.761**	0.364	< 0.001
AD2	− 0.182	0.214	0.395	**− 0.532**	0.231	0.022	**− 0.872**	0.267	0.001	**− 1.293**	0.337	< 0.001
AD3	**− 0.539**	0.218	0.013	**− 1.028**	0.247	< 0.001	**− 0.670**	0.236	0.005	**− 2.108**	0.407	< 0.001
AD4	**− 1.597**	0.234	< 0.001	**− 1.313**	0.278	< 0.001	**− 2.001**	0.368	< 0.001	**− 3.638**	0.477	< 0.001
AD5	**− 2.290**	0.266	< 0.001	**− 1.619**	0.270	< 0.001	**− 1.599**	0.342	< 0.001	**− 4.140**	0.588	< 0.001
Significant interaction terms (*n*)			12			15			13			15
AIC			4112			3752			3875			3999
BIC			4360			3999			4122			4247
LL			− 2016			− 1836			− 1898			− 1959

*AD* anxiety/depression, *AIC* Akaike information criterion, *BIC* Bayesian information criterion, *Coef* coefficient estimate, *Gen-dev* generator-developed designs [11], *LL* log-likelihood, *MO* mobility, *Ngene* modified Fedorov designs constructed in Ngene [9],* Obs* number of observations, *P* *p*-value, *PD* pain/discomfort, *R* Bayesian D-efficient design algorithm implemented in R by Oppe and van Hout [14] based on Rose et al. [15], *SAS* modified Fedorov designs constructed in SAS [10], *SC* self-care, *SE* standard error, *UA* usual activities

Significant interaction terms are in bold

**Fig. 2:**
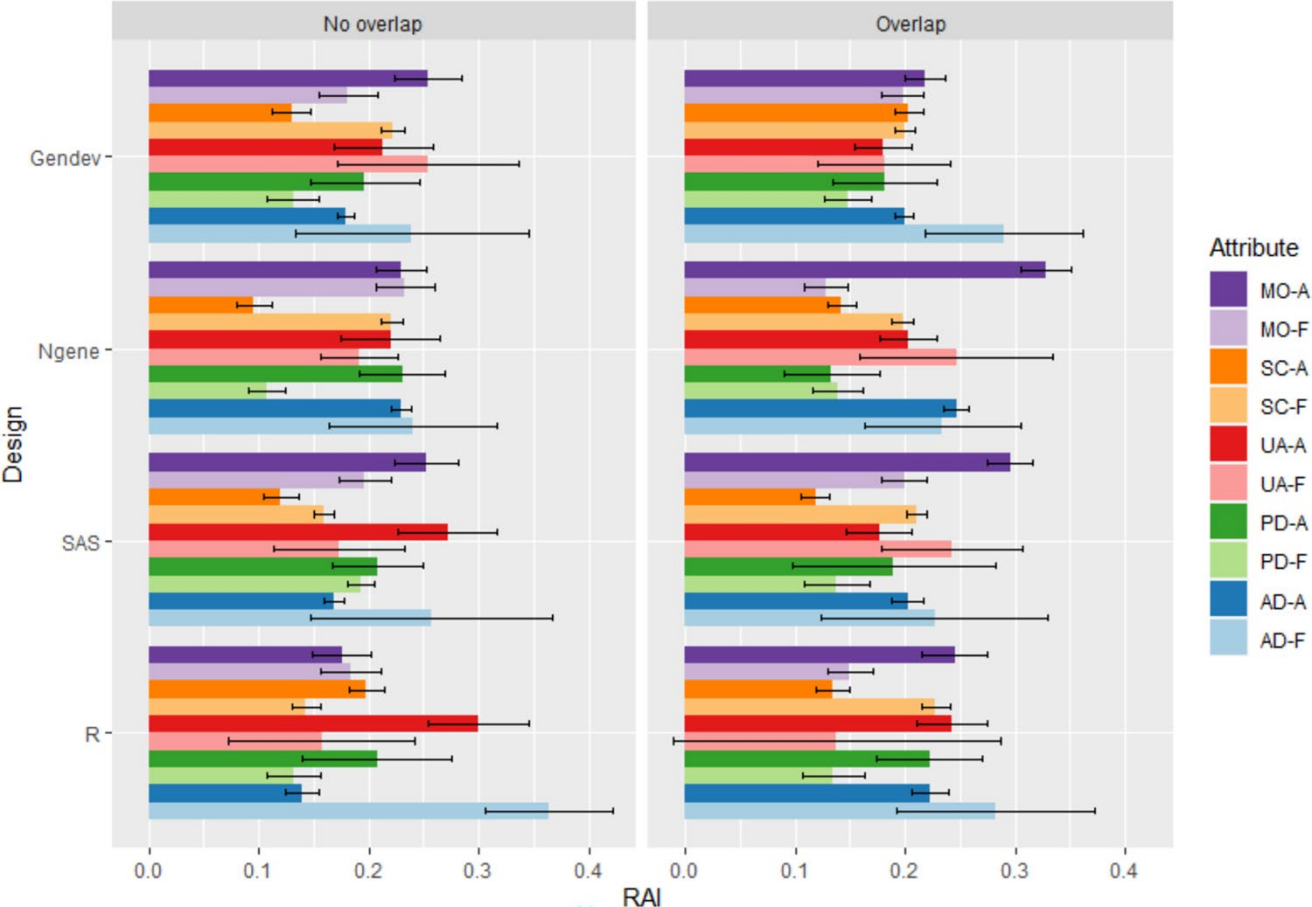
Relative attribute importance (RAI). *A* all respondents, *AD* anxiety/depression, *F* full attenders, *Gen-dev* generator-developed designs [11], *MO* mobility, *Ngene* modified Fedorov designs constructed in Ngene [9], *PD* pain/discomfort, *R* Bayesian D-efficient design algorithm implemented in R by Oppe and van Hout [14] based on Rose et al. [15], *SAS* modified Fedorov designs constructed in SAS [10], *SC* self-care, *UA* usual activities

MNLs with main effects only were then fitted using data from both all respondents and full attenders only (unanchored and anchored MNL results can be found in ESM-B). Figure 2 shows the RAI scores derived from these MNL results. The RAI scores for self-care, usual activities, and pain/discomfort varied across designs, but there was no clear pattern. A notable observation was found when comparing RAI for all respondents versus full attenders, especially in the dimensions of mobility and anxiety/depression. Mobility consistently showed higher importance when using the data before exclusion as opposed to the data from only full attenders. Conversely, anxiety/depression had greater importance assigned to this dimension among full attenders than among their counterparts.

### External Validity of the ECLC Model

Table 4 summarises the indicators of external validity across the three attendance classes identified by the ECLC model. Respondents classified as non-attenders had the highest overall rate of straightlining (3.9%), whereas partial attenders showed moderate levels (1.2%) and full attenders the lowest (0.4%). Median completion times mirrored this pattern: non-attenders spent the least time on the choice tasks (3.2 min) and on the entire survey (7.6 min), followed by partial attenders (5.0 and 8.7 min, respectively), and finally full attenders, who spent the most time on both the tasks (6.3 min) and the survey (10.2 min).

**Table 4:** Straightlining, completion time, and feedback questions across three attendance classes

	Full attendance (*N* = 493)	Partial attendance (*n* = 705)	Non-attendance (*n* = 234)
Straightlining			
Left-most option	2 (0.4)	8 (1.1)	6 (2.6)
Right-most option	0 (0.0)	1 (0.1)	3 (1.3)
Total	2 (0.4)	9 (1.2)	9 (3.9)
Completion time			
DCE choice tasks	6.3	5.0	3.2
Entire survey	10.2	8.7	7.6
Feedback question^a^			
Task difficult^b^	45 (9.7)	87 (12.8)	39 (17.3)
Difficult to tell difference^c^	52 (11.2)	124 (18.3)	60 (26.6)
Difficult to imagine^d^	84 (18.1)	138 (20.4)	51 (22.7)
Consider whole description^e^	386 (82.5)	483 (71.3)	125 (55.6)

Data are presented as minutes or as *N* (%)

*DCE* discrete choice experiment

^a^These questions were optional, and the reported percentages were calculated only among respondents who provided answers

^b^Percentage of respondents who selected ‘strongly agree’ or ‘agree’ for the question, "I found the tasks difficult"

^c^Percentage of respondents who selected ‘strongly agree’ or ‘agree’ for the question, "I found it difficult to tell the difference between the descriptions"

^d^Percentage of respondents who selected ‘strongly agree’ or ‘agree’ to the question, "I found it difficult to imagine the scenarios"

^e^Percentage of respondents who selected ‘strongly agree’ or ‘agree’ to the question, "I considered the entire description"

Feedback question responses aligned with these differences in response behaviour. Non-attenders were more likely to report that the tasks were difficult (17.3% vs. 12.8% of partial attenders and 9.7% of full attenders) and to find it difficult to tell the difference between the options (26.6% vs. 18.3% and 11.2%, respectively). In addition, only 55.6% of non-attenders strongly agreed or agreed that they considered the entire description when making their choices, in contrast to 71.3% of partial attenders and 82.5% of full attenders. These findings provide strong evidence for the external validity of the ECLC model’s classification of full attenders, partial attenders, and non-attenders, reflecting correspondingly high, moderate, and low levels of engagement and attentiveness.

In addition, demographic characteristics across the three attendance classes are presented in Table 3 in ESM-B. Non-attenders were more likely to be male, younger, and single than both full or partial attenders. No apparent differences were observed in education, household income, or region among the three classes.

## Discussion

Our analysis revealed significant variations in attribute attendance across diverse design construction methods. Moreover, the incorporation of attribute overlap yielded a significant enhancement in attribute attendance levels. In particular, modified Fedorov designs (implemented in Ngene and SAS) with attribute overlap had the highest attribute attendance rates, supporting the use of modified Fedorov designs with attribute overlap to enhance respondent engagement.

The ANA probabilities within designs without overlap in this study are in line with the results from the comparison study conducted by Iles and Rose [5] but inconsistent with the findings of Yao et al. [6]. Iles and Rose [5] found a higher percentage of ANA in answering efficient design surveys than with orthogonal designs, among both illiterate and literate respondents. However, Yao et al. [6] reported conflicting findings: that the likelihood of belonging to the class with full attendance was greater in efficient designs than in generator-developed designs. The disparate findings may be explained by the different levels of perceived difficulty of the survey. Iles and Rose [5] focused on preferences for healthcare providers among illiterate and literate respondents in India, whereas Yao et al. [6] recruited respondents from the general New Zealand population to answer questions about threatened species in planted forests. Several factors may contribute to the variance in full attendance rates, such as perceived difficulty levels, familiarity with the topic of the survey, and population characteristics. Exploring the impact of these factors on attribute attendance is a valuable potential area for future research.

Another important finding is that attribute overlap substantially enhanced the level of attribute attendance across the dimensions of the EQ-5D-5L, regardless of the methods employed in design construction. This finding aligns with the conclusions drawn by Jonker et al. [12], which suggested that attribute overlap leads to a rise in the average number of attended attributes from two to three. In our study, when examining the Ngene design, we observed that attribute overlap increases the full attendance rate significantly from 24.6% to 54.2%. This indicates that, even with the implementation of attribute overlap, approximately 50% of the respondents did not engage with at least one dimension of the EQ-5D-5L despite the use of attribute overlap. ANA analysis can serve as a useful quality-control tool for the inclusion and exclusion of respondents in data analysis. Ideally, the analysis should exclude respondents who did not attend to any attributes, as their choices are likely to be driven by heuristic decision-making rather than reflective of their true preferences. However, partial non-attendance may indicate either a lack of attention or a deliberate decision reflecting genuine indifference. Additional robustness checks may be warranted, particularly for respondents who focus exclusively on a single attribute.

The consideration of ANA has an influence on the coefficients of the EQ-5D-5L in health state valuations. This impact was more pronounced within the mobility and anxiety/depression dimensions. Upon ANA adjustment, the utility decrement in the mobility dimension decreased, whereas the decrements associated with anxiety/depression increased. Although this study did not directly test the order effects, it may be that respondents, especially partial attenders, prioritised dimensions presented earlier in the sequence of mobility, self-care, usual activities, pain/discomfort, and anxiety/depression. This possibility is supported by previous research indicating that the dimension order has some influence on the valuation of the EQ-5D-5L, using methods such as DCE_TTO_ [21], time trade-off (TTO) [22], and DCE [22]. In contrast, Mulhern et al. [23] observed no significant impact of dimension order in DCEs with duration. This discrepancy highlights the potential variability in how different preference-elicitation techniques may interact with dimension ordering. Therefore, further research is warranted to dissect the influence of dimension order on EQ-5D-5L valuations across various elicitation methods.

Preference heterogeneity is another possible explanation of the impact of ANA on the coefficients of the EQ-5D-5L. Within the framework of ECLC models, preference homogeneity among respondents is assumed. However, ANA may occur when respondents do not consider attributes because they are not important to them or arise from respondents simplifying choice tasks by ignoring attributes as a type of decision heuristic. Indeed, the impact of ANA on the valuation of EQ-5D-5L varied significantly between these two ANA assumptions [16]. A substantial impact of ANA on estimates was observed when assuming decision heuristics for ANA, whereas the impact was less pronounced under the preference-based explanation of ANA [16]. In our study, respondents placing lower importance on a particular attribute might be inaccurately classified as non-attenders. In alignment with a study by Hole et al. [24], the findings may reflect the upper limits relating to ANA within this dataset.

## Conclusion

Attribute attendance varied across the different design construction methods, and the implementation of attribute overlap significantly improved attendance rates. Modified Fedorov designs implemented in Ngene or SAS with attribute overlap are recommended to reduce ANA and enhance respondent engagement in DCEs. ANA models can be used to exclude respondents who did not attend to any attributes and to identify partial attenders for further robustness checks, improving the quality of data for analysis.

## Supplementary Information

Below is the link to the electronic supplementary material.

Supplementary file1 (DOCX 58 KB)

## Declarations

### Data Availability

The data that support the findings of this study are available from the Centre for Health Economics Research and Evaluation, but restrictions that were applied in relation to the ethics of the current study apply to the availability of these data, so they are not publicly available. However, the data are available from the authors upon reasonable request and with the permission of the Centre for Health Economics Research and Evaluation.
