# Supplementary material for: How do Design Characteristics Affect Respondent Engagement? Assessing Attribute Non-attendance in Discrete Choice Experiments Valuing the EQ-5D-5L
Source: Patient. 2025 Mar 15;18(4):329–41. doi: 10.1007/s40271-025-00735-9 (PMC12170736; doi:10.1007/s40271-025-00735-9)
Supplement: Supplementary file 1 — Supplementary file1 (DOCX 58 KB) [file 40271_2025_735_MOESM1_ESM.docx]

# Appendices

## Appendix A: Specific requirement for level balance in R designs

The designs were generated randomly, with the constraints that no pair could be repeated (order of options within the choice sets is not relevant), no choice sets could contain a dominated option and the designs had to satisfy a pre-specified criteria for level balance. Level balance is measured by the value of

$$v= \sum_{a=1}^{5} \sum_{i=1}^{4} \sum_{j=i+1}^{5} \left( r_{ai}-r_{aj} \right)^{2}$$

where *r_ai_* is the number of times that level *i* appears in attribute *a*. As 100 levels of each attribute appear in the design, the largest value that *v* can take is 200,000 when only one level appears in each of the five attributes (and of course then all of the options in all of the choice sets are the same and the design is useless by any measure). When all the levels of all of the attributes appear equally often then *v* = 0. The second smallest possible value of *v* is 10 (when the levels of four of the attributes are equally replicated and the fifth attribute has replications of 20, 20, 20, 21 and 19 in some order). Any designs in which *v* exceeds 900 were excluded.

Designs were constructed assuming a normal prior distribution centred at 0 for the non-informative prior value, with the standard error assumed to be 0.05. The number of designs tested depended on both the assumed prior and level of overlap. For each design, the D-Error was calculated for a maximum of 20,000 (this number was arbitrarily chosen) designs. The D-Error is calculated by scaling the determinant of the variance covariance matrix by the number of parameters in the model. In total about 970,000 designs were created for each of the designs with no overlap (and about 950,000 were rejected due to level balance being greater than 900) and about 5,125,193 for each of the designs with overlap on two attributes (of which about 5,100,000 were rejected due to level balance being greater than 900).

## Appendix B

Table 1a. MNL estimates derived from designs with no overlap

|  | All respondents | | | | | | | | Full attenders only | | | | | | | |
| --- | --- | --- | --- | --- | --- | --- | --- | --- | --- | --- | --- | --- | --- | --- | --- | --- |
|  | Gendev | | Ngene | | SAS | | R | | Gendev | | Ngene | | SAS | | R | |
|  | Coef | SE | Coef | SE | Coef | SE | Coef | SE | Coef | SE | Coef | SE | Coef | SE | Coef | SE |
| MO2 | -0.292 | 0.076 | -0.095 | 0.076 | -0.203 | 0.077 | -0.022 | 0.105 | -0.480 | 0.210 | -0.001 | 0.218 | -0.539 | 0.248 | -0.122 | 0.366 |
| MO3 | -0.282 | 0.078 | -0.279 | 0.080 | -0.279 | 0.079 | -0.159 | 0.085 | -0.920 | 0.230 | -0.866 | 0.228 | -0.363 | 0.285 | -0.924 | 0.241 |
| MO4 | -0.728 | 0.080 | -0.847 | 0.084 | -0.862 | 0.083 | -0.550 | 0.098 | -1.968 | 0.261 | -1.621 | 0.237 | -1.693 | 0.315 | -1.974 | 0.365 |
| MO5 | -1.107 | 0.081 | -1.226 | 0.091 | -1.199 | 0.082 | -0.828 | 0.099 | -2.848 | 0.258 | -2.506 | 0.272 | -2.423 | 0.312 | -2.451 | 0.351 |
| SC2 | -0.167 | 0.076 | -0.292 | 0.076 | -0.185 | 0.079 | -0.100 | 0.095 | -0.443 | 0.204 | -0.628 | 0.214 | -0.510 | 0.268 | -0.792 | 0.292 |
| SC3 | -0.201 | 0.079 | -0.348 | 0.078 | -0.185 | 0.078 | -0.075 | 0.092 | -0.360 | 0.238 | -1.116 | 0.208 | -0.557 | 0.270 | -1.240 | 0.320 |
| SC4 | -0.558 | 0.079 | -0.922 | 0.082 | -0.675 | 0.079 | -0.548 | 0.092 | -1.670 | 0.244 | -2.160 | 0.243 | -2.345 | 0.255 | -2.332 | 0.343 |
| SC5 | -0.793 | 0.082 | -0.922 | 0.085 | -0.937 | 0.080 | -0.903 | 0.109 | -2.340 | 0.277 | -2.110 | 0.254 | -2.635 | 0.293 | -2.858 | 0.366 |
| UA2 | -0.153 | 0.076 | 0.059 | 0.081 | -0.126 | 0.078 | -0.155 | 0.100 | -0.234 | 0.202 | 0.126 | 0.223 | -0.549 | 0.260 | -0.230 | 0.305 |
| UA3 | -0.203 | 0.080 | -0.108 | 0.085 | -0.207 | 0.079 | -0.187 | 0.099 | -0.427 | 0.247 | -0.242 | 0.243 | -0.739 | 0.258 | -0.379 | 0.293 |
| UA4 | -0.324 | 0.080 | -0.395 | 0.078 | -0.368 | 0.080 | -0.577 | 0.090 | -0.826 | 0.251 | -1.168 | 0.217 | -1.852 | 0.329 | -1.772 | 0.288 |
| UA5 | -0.551 | 0.081 | -0.574 | 0.075 | -0.508 | 0.084 | -0.833 | 0.097 | -1.728 | 0.250 | -1.510 | 0.210 | -2.457 | 0.351 | -2.203 | 0.287 |
| PD2 | -0.150 | 0.073 | -0.183 | 0.081 | -0.107 | 0.078 | 0.042 | 0.103 | -0.043 | 0.195 | -0.455 | 0.215 | -0.223 | 0.252 | -0.149 | 0.333 |
| PD3 | -0.343 | 0.075 | -0.404 | 0.082 | -0.145 | 0.078 | -0.043 | 0.116 | -0.368 | 0.200 | -0.916 | 0.217 | -0.378 | 0.269 | -0.542 | 0.345 |
| PD4 | -0.669 | 0.074 | -0.802 | 0.082 | -0.608 | 0.075 | -0.518 | 0.085 | -1.558 | 0.197 | -2.217 | 0.247 | -1.782 | 0.276 | -1.860 | 0.315 |
| PD5 | -0.941 | 0.079 | -0.964 | 0.086 | -0.743 | 0.079 | -0.747 | 0.107 | -2.409 | 0.241 | -2.412 | 0.261 | -2.506 | 0.395 | -2.303 | 0.344 |
| AD2 | -0.013 | 0.073 | -0.199 | 0.077 | -0.384 | 0.075 | -0.065 | 0.097 | -0.134 | 0.197 | -0.653 | 0.215 | -1.178 | 0.254 | -1.193 | 0.320 |
| AD3 | -0.269 | 0.074 | -0.213 | 0.080 | -0.443 | 0.077 | -0.433 | 0.113 | -0.675 | 0.201 | -1.108 | 0.230 | -1.044 | 0.220 | -2.289 | 0.388 |
| AD4 | -0.847 | 0.075 | -0.874 | 0.086 | -0.944 | 0.085 | -0.998 | 0.108 | -2.180 | 0.219 | -2.046 | 0.262 | -2.794 | 0.356 | -4.254 | 0.463 |
| AD5 | -0.896 | 0.079 | -1.053 | 0.082 | -0.969 | 0.082 | -1.157 | 0.102 | -2.863 | 0.252 | -2.449 | 0.254 | -2.444 | 0.330 | -4.913 | 0.578 |
| AIC |  | 4270 |  | 3920 |  | 4048 |  | 4192 |  | 806 |  | 720 |  | 589 |  | 634 |
| BIC |  | 4394 |  | 4043 |  | 4171 |  | 4316 |  | 904 |  | 819 |  | 683 |  | 730 |
| LL |  | -2115 |  | -1940 |  | -2004 |  | -2076 |  | -383 |  | -340 |  | -275 |  | -297 |

Note: Gen-dev = generator-developed designs (Street & Burgess, 2007); Ngene = modified Fedorov designs constructed in Ngene (ChoiceMetrics, 2014); SAS = modified Fedorov designs constructed in SAS (Kuhfeld, 2010); R = Bayesian D-efficient design algorithm implemented in R by Oppe & van Hout (2017) based on Rose, Scarpa & Bliemer (2009)

Table 1b. MNL estimates derived from designs with overlap

|  | All respondents | | | | | | | | Full attenders only | | | | | | | |
| --- | --- | --- | --- | --- | --- | --- | --- | --- | --- | --- | --- | --- | --- | --- | --- | --- |
|  | Gendev | | Ngene | | SAS | | R | | Gendev | | Ngene | | SAS | | R | |
|  | Coef | SE | Coef | SE | Coef | SE | Coef | SE | Coef | SE | Coef | SE | Coef | SE | Coef | SE |
| MO2 | -0.240 | 0.104 | -0.557 | 0.097 | -0.467 | 0.103 | -0.429 | 0.105 | -0.328 | 0.235 | -0.849 | 0.157 | -0.679 | 0.176 | -0.343 | 0.254 |
| MO3 | -0.582 | 0.130 | -0.564 | 0.108 | -0.833 | 0.110 | -0.305 | 0.101 | -1.168 | 0.299 | -0.887 | 0.174 | -1.378 | 0.224 | -0.621 | 0.266 |
| MO4 | -1.166 | 0.135 | -1.142 | 0.120 | -1.185 | 0.106 | -0.935 | 0.112 | -2.057 | 0.314 | -1.886 | 0.195 | -2.684 | 0.239 | -1.617 | 0.259 |
| MO5 | -1.607 | 0.117 | -1.705 | 0.120 | -1.959 | 0.125 | -1.197 | 0.112 | -3.100 | 0.287 | -2.570 | 0.207 | -3.049 | 0.269 | -2.300 | 0.291 |
| SC2 | -0.059 | 0.106 | -0.004 | 0.101 | -0.310 | 0.114 | -0.104 | 0.112 | -0.282 | 0.245 | -0.111 | 0.175 | -0.196 | 0.228 | -1.291 | 0.291 |
| SC3 | -0.524 | 0.131 | -0.102 | 0.111 | -0.289 | 0.106 | -0.123 | 0.127 | -1.163 | 0.296 | -0.453 | 0.196 | -0.646 | 0.187 | -0.965 | 0.302 |
| SC4 | -1.108 | 0.133 | -0.515 | 0.109 | -1.037 | 0.115 | -0.540 | 0.132 | -2.141 | 0.307 | -1.118 | 0.194 | -2.227 | 0.233 | -2.230 | 0.368 |
| SC5 | -1.572 | 0.115 | -0.929 | 0.106 | -1.368 | 0.117 | -0.988 | 0.128 | -3.096 | 0.284 | -1.662 | 0.208 | -3.016 | 0.261 | -3.400 | 0.428 |
| UA2 | -0.278 | 0.076 | 0.073 | 0.094 | 0.155 | 0.115 | -0.146 | 0.120 | -0.520 | 0.180 | -0.030 | 0.150 | 0.295 | 0.248 | -0.498 | 0.272 |
| UA3 | -0.508 | 0.076 | -0.385 | 0.103 | -0.238 | 0.108 | -0.171 | 0.117 | -0.718 | 0.191 | -0.912 | 0.177 | -0.293 | 0.227 | -0.460 | 0.268 |
| UA4 | -1.138 | 0.081 | -0.554 | 0.095 | -0.617 | 0.109 | -0.497 | 0.124 | -1.956 | 0.230 | -1.136 | 0.169 | -1.775 | 0.204 | -1.736 | 0.323 |
| UA5 | -1.424 | 0.082 | -0.854 | 0.098 | -1.080 | 0.104 | -0.749 | 0.154 | -2.233 | 0.205 | -1.405 | 0.161 | -2.650 | 0.214 | -2.076 | 0.373 |
| PD2 | -0.283 | 0.104 | -0.235 | 0.112 | -0.021 | 0.103 | -0.142 | 0.114 | -0.526 | 0.251 | -0.509 | 0.185 | -0.166 | 0.192 | -0.086 | 0.296 |
| PD3 | -0.368 | 0.133 | -0.241 | 0.102 | -0.587 | 0.103 | -0.391 | 0.116 | -0.749 | 0.320 | -0.813 | 0.175 | -1.129 | 0.200 | -1.064 | 0.271 |
| PD4 | -1.149 | 0.139 | -0.793 | 0.113 | -1.212 | 0.104 | -1.073 | 0.102 | -2.610 | 0.375 | -2.110 | 0.205 | -2.488 | 0.220 | -3.163 | 0.302 |
| PD5 | -1.498 | 0.122 | -1.123 | 0.094 | -1.340 | 0.113 | -1.059 | 0.133 | -3.446 | 0.331 | -2.763 | 0.193 | -2.719 | 0.249 | -3.572 | 0.408 |
| AD2 | -0.202 | 0.114 | -0.159 | 0.133 | -0.014 | 0.100 | 0.129 | 0.148 | -0.625 | 0.303 | -0.104 | 0.233 | -0.662 | 0.211 | -0.449 | 0.384 |
| AD3 | -0.501 | 0.134 | -0.578 | 0.129 | -0.344 | 0.096 | -0.373 | 0.125 | -1.769 | 0.358 | -1.024 | 0.200 | -1.174 | 0.199 | -1.467 | 0.319 |
| AD4 | -1.020 | 0.136 | -0.711 | 0.128 | -1.059 | 0.104 | -1.300 | 0.133 | -3.593 | 0.373 | -2.174 | 0.223 | -2.669 | 0.257 | -4.283 | 0.470 |
| AD5 | -1.244 | 0.120 | -1.011 | 0.107 | -1.235 | 0.112 | -1.287 | 0.121 | -4.687 | 0.408 | -2.560 | 0.195 | -2.783 | 0.289 | -3.813 | 0.419 |
| AIC |  | 4136 |  | 4067 |  | 3751 |  | 4315 |  | 915 |  | 1666 |  | 1382 |  | 973 |
| BIC |  | 4260 |  | 4191 |  | 3874 |  | 4439 |  | 1016 |  | 1778 |  | 1494 |  | 1072 |
| LL |  | -2048 |  | -2014 |  | -1855 |  | -2138 |  | -438 |  | -813 |  | -671 |  | -466 |

Note: Gen-dev = generator-developed designs (Street & Burgess, 2007); Ngene = modified Fedorov designs constructed in Ngene (ChoiceMetrics, 2014); SAS = modified Fedorov designs constructed in SAS (Kuhfeld, 2010); R = Bayesian D-efficient design algorithm implemented in R by Oppe & van Hout (2017) based on Rose, Scarpa & Bliemer (2009)

Table 2a. Anchored MNL estimates derived from designs with no overlap

|  | All respondents | | | | | | | | Full attenders only | | | | | | | |
| --- | --- | --- | --- | --- | --- | --- | --- | --- | --- | --- | --- | --- | --- | --- | --- | --- |
|  | Gendev | | Ngene | | SAS | | R | | Gendev | | Ngene | | SAS | | R | |
|  | Coef | SE | Coef | SE | Coef | SE | Coef | SE | Coef | SE | Coef | SE | Coef | SE | Coef | SE |
| MO2 | -0.020 | 0.016 | -0.047 | 0.018 | -0.005 | 0.023 |  | 0.017 | 0.000 | 0.020 | -0.043 | 0.020 | -0.008 | 0.025 | -0.020 | 0.016 |
| MO3 | -0.059 | 0.017 | -0.064 | 0.018 | -0.035 | 0.019 | -0.075 | 0.019 | -0.079 | 0.021 | -0.029 | 0.023 | -0.063 | 0.016 | -0.059 | 0.017 |
| MO4 | -0.179 | 0.018 | -0.198 | 0.019 | -0.123 | 0.022 | -0.161 | 0.021 | -0.148 | 0.022 | -0.136 | 0.025 | -0.134 | 0.025 | -0.179 | 0.018 |
| MO5 | -0.259 | 0.019 | -0.275 | 0.019 | -0.185 | 0.022 | -0.234 | 0.021 | -0.228 | 0.025 | -0.194 | 0.025 | -0.166 | 0.024 | -0.259 | 0.019 |
| SC2 | -0.062 | 0.016 | -0.043 | 0.018 | -0.022 | 0.021 | -0.036 | 0.017 | -0.057 | 0.019 | -0.041 | 0.022 | -0.054 | 0.020 | -0.062 | 0.016 |
| SC3 | -0.073 | 0.016 | -0.042 | 0.018 | -0.017 | 0.021 | -0.030 | 0.020 | -0.102 | 0.019 | -0.045 | 0.022 | -0.084 | 0.022 | -0.073 | 0.016 |
| SC4 | -0.195 | 0.017 | -0.155 | 0.018 | -0.123 | 0.021 | -0.137 | 0.020 | -0.197 | 0.022 | -0.188 | 0.020 | -0.158 | 0.023 | -0.195 | 0.017 |
| SC5 | -0.195 | 0.018 | -0.215 | 0.018 | -0.202 | 0.024 | -0.192 | 0.023 | -0.192 | 0.023 | -0.211 | 0.023 | -0.194 | 0.025 | -0.195 | 0.018 |
| UA2 | 0.012 | 0.017 | -0.029 | 0.018 | -0.035 | 0.022 | -0.019 | 0.017 | 0.011 | 0.020 | -0.044 | 0.021 | -0.016 | 0.021 | 0.012 | 0.017 |
| UA3 | -0.023 | 0.018 | -0.048 | 0.018 | -0.042 | 0.022 | -0.035 | 0.020 | -0.022 | 0.022 | -0.059 | 0.021 | -0.026 | 0.020 | -0.023 | 0.018 |
| UA4 | -0.083 | 0.016 | -0.084 | 0.018 | -0.129 | 0.020 | -0.068 | 0.021 | -0.106 | 0.020 | -0.149 | 0.026 | -0.120 | 0.020 | -0.083 | 0.016 |
| UA5 | -0.121 | 0.016 | -0.117 | 0.019 | -0.187 | 0.022 | -0.142 | 0.020 | -0.137 | 0.019 | -0.197 | 0.028 | -0.150 | 0.019 | -0.121 | 0.016 |
| PD2 | -0.039 | 0.017 | -0.025 | 0.018 | 0.009 | 0.023 | -0.004 | 0.016 | -0.041 | 0.020 | -0.018 | 0.020 | -0.010 | 0.023 | -0.039 | 0.017 |
| PD3 | -0.085 | 0.017 | -0.033 | 0.018 | -0.010 | 0.026 | -0.030 | 0.016 | -0.083 | 0.020 | -0.030 | 0.022 | -0.037 | 0.023 | -0.085 | 0.017 |
| PD4 | -0.169 | 0.017 | -0.140 | 0.017 | -0.116 | 0.019 | -0.128 | 0.016 | -0.202 | 0.022 | -0.143 | 0.022 | -0.126 | 0.021 | -0.169 | 0.017 |
| PD5 | -0.203 | 0.018 | -0.171 | 0.018 | -0.167 | 0.024 | -0.198 | 0.020 | -0.220 | 0.024 | -0.201 | 0.032 | -0.156 | 0.023 | -0.203 | 0.018 |
| AD2 | -0.042 | 0.016 | -0.088 | 0.017 | -0.015 | 0.022 | -0.011 | 0.016 | -0.059 | 0.020 | -0.094 | 0.020 | -0.081 | 0.022 | -0.042 | 0.016 |
| AD3 | -0.045 | 0.017 | -0.102 | 0.018 | -0.097 | 0.025 | -0.055 | 0.017 | -0.101 | 0.021 | -0.084 | 0.018 | -0.155 | 0.026 | -0.045 | 0.017 |
| AD4 | -0.184 | 0.018 | -0.217 | 0.019 | -0.223 | 0.024 | -0.179 | 0.018 | -0.186 | 0.024 | -0.224 | 0.029 | -0.289 | 0.031 | -0.184 | 0.018 |
| AD5 | -0.222 | 0.017 | -0.222 | 0.019 | -0.259 | 0.023 | -0.235 | 0.021 | -0.223 | 0.023 | -0.196 | 0.026 | -0.334 | 0.039 | -0.222 | 0.017 |
| AIC |  | 4270 |  | 3920 |  | 4048 |  | 4192 |  | 806 |  | 720 |  | 589 |  | 634 |
| BIC |  | 4394 |  | 4043 |  | 4171 |  | 4316 |  | 904 |  | 819 |  | 683 |  | 730 |
| LL |  | -2115 |  | -1940 |  | -2004 |  | -2076 |  | -383 |  | -340 |  | -275 |  | -297 |

Note: Gen-dev = generator-developed designs (Street & Burgess, 2007); Ngene = modified Fedorov designs constructed in Ngene (ChoiceMetrics, 2014); SAS = modified Fedorov designs constructed in SAS (Kuhfeld, 2010); R = Bayesian D-efficient design algorithm implemented in R by Oppe & van Hout (2017) based on Rose, Scarpa & Bliemer (2009)

Table 2b. Anchored MNL estimates derived from designs with overlap

|  | All respondents | | | | | | | | Full attenders only | | | | | | | |
| --- | --- | --- | --- | --- | --- | --- | --- | --- | --- | --- | --- | --- | --- | --- | --- | --- |
|  | Gendev | | Ngene | | SAS | | R | | Gendev | | Ngene | | SAS | | R | |
|  | Coef | SE | Coef | SE | Coef | SE | Coef | SE | Coef | SE | Coef | SE | Coef | SE | Coef | SE |
| MO2 | -0.099 | 0.017 | -0.067 | 0.015 | -0.081 | 0.020 | -0.020 | 0.014 | -0.077 | 0.014 | -0.048 | 0.012 | -0.023 | 0.017 | -0.099 | 0.017 |
| MO3 | -0.100 | 0.019 | -0.119 | 0.016 | -0.058 | 0.019 | -0.071 | 0.018 | -0.081 | 0.016 | -0.097 | 0.016 | -0.041 | 0.018 | -0.100 | 0.019 |
| MO4 | -0.203 | 0.021 | -0.170 | 0.015 | -0.177 | 0.021 | -0.124 | 0.019 | -0.172 | 0.018 | -0.189 | 0.017 | -0.107 | 0.017 | -0.203 | 0.021 |
| MO5 | -0.303 | 0.021 | -0.281 | 0.018 | -0.227 | 0.021 | -0.187 | 0.017 | -0.234 | 0.019 | -0.214 | 0.019 | -0.152 | 0.019 | -0.303 | 0.021 |
| SC2 | -0.001 | 0.018 | -0.044 | 0.016 | -0.020 | 0.021 | -0.017 | 0.015 | -0.010 | 0.016 | -0.014 | 0.016 | -0.085 | 0.019 | -0.001 | 0.018 |
| SC3 | -0.018 | 0.020 | -0.041 | 0.015 | -0.023 | 0.024 | -0.070 | 0.018 | -0.041 | 0.018 | -0.045 | 0.013 | -0.064 | 0.020 | -0.018 | 0.020 |
| SC4 | -0.092 | 0.019 | -0.149 | 0.016 | -0.102 | 0.025 | -0.129 | 0.019 | -0.102 | 0.018 | -0.157 | 0.016 | -0.147 | 0.024 | -0.092 | 0.019 |
| SC5 | -0.165 | 0.019 | -0.196 | 0.017 | -0.187 | 0.024 | -0.187 | 0.017 | -0.152 | 0.019 | -0.212 | 0.018 | -0.224 | 0.028 | -0.165 | 0.019 |
| UA2 | 0.013 | 0.017 | 0.022 | 0.017 | -0.028 | 0.023 | -0.031 | 0.011 | -0.003 | 0.014 | 0.021 | 0.017 | -0.033 | 0.018 | 0.013 | 0.017 |
| UA3 | -0.068 | 0.018 | -0.034 | 0.015 | -0.032 | 0.022 | -0.043 | 0.012 | -0.083 | 0.016 | -0.021 | 0.016 | -0.030 | 0.018 | -0.068 | 0.018 |
| UA4 | -0.099 | 0.017 | -0.088 | 0.016 | -0.094 | 0.024 | -0.118 | 0.014 | -0.104 | 0.015 | -0.125 | 0.014 | -0.115 | 0.021 | -0.099 | 0.017 |
| UA5 | -0.152 | 0.018 | -0.155 | 0.015 | -0.142 | 0.029 | -0.135 | 0.012 | -0.128 | 0.015 | -0.186 | 0.015 | -0.137 | 0.025 | -0.152 | 0.018 |
| PD2 | -0.042 | 0.020 | -0.003 | 0.015 | -0.027 | 0.022 | -0.032 | 0.015 | -0.046 | 0.017 | -0.012 | 0.014 | -0.006 | 0.020 | -0.042 | 0.020 |
| PD3 | -0.043 | 0.018 | -0.084 | 0.015 | -0.074 | 0.022 | -0.045 | 0.019 | -0.074 | 0.016 | -0.079 | 0.014 | -0.070 | 0.018 | -0.043 | 0.018 |
| PD4 | -0.141 | 0.020 | -0.174 | 0.015 | -0.203 | 0.019 | -0.158 | 0.023 | -0.192 | 0.019 | -0.175 | 0.015 | -0.209 | 0.020 | -0.141 | 0.020 |
| PD5 | -0.200 | 0.017 | -0.192 | 0.016 | -0.201 | 0.025 | -0.208 | 0.020 | -0.252 | 0.018 | -0.191 | 0.017 | -0.236 | 0.027 | -0.200 | 0.017 |
| AD2 | -0.028 | 0.024 | -0.002 | 0.014 | 0.024 | 0.028 | -0.038 | 0.018 | -0.009 | 0.021 | -0.047 | 0.015 | -0.030 | 0.025 | -0.028 | 0.024 |
| AD3 | -0.103 | 0.023 | -0.049 | 0.014 | -0.071 | 0.024 | -0.107 | 0.022 | -0.093 | 0.018 | -0.083 | 0.014 | -0.097 | 0.021 | -0.103 | 0.023 |
| AD4 | -0.126 | 0.023 | -0.152 | 0.015 | -0.246 | 0.025 | -0.217 | 0.023 | -0.198 | 0.020 | -0.188 | 0.018 | -0.282 | 0.031 | -0.126 | 0.023 |
| AD5 | -0.180 | 0.019 | -0.177 | 0.016 | -0.244 | 0.023 | -0.283 | 0.025 | -0.234 | 0.018 | -0.196 | 0.020 | -0.251 | 0.028 | -0.180 | 0.019 |
| AIC |  | 4136 |  | 4067 |  | 3751 |  | 4315 |  | 915 |  | 1666 |  | 1382 |  | 973 |
| BIC |  | 4260 |  | 4191 |  | 3874 |  | 4439 |  | 1016 |  | 1778 |  | 1494 |  | 1072 |
| LL |  | -2048 |  | -2014 |  | -1855 |  | -2138 |  | -438 |  | -813 |  | -671 |  | -466 |

Note: Gen-dev = generator-developed designs (Street & Burgess, 2007); Ngene = modified Fedorov designs constructed in Ngene (ChoiceMetrics, 2014); SAS = modified Fedorov designs constructed in SAS (Kuhfeld, 2010); R = Bayesian D-efficient design algorithm implemented in R by Oppe & van Hout (2017) based on Rose, Scarpa & Bliemer (2009)

Table 3. Demographics across the three classes

|  |  | Full (%) | Partial (%) | None (%) |
| --- | --- | --- | --- | --- |
| Gender | Male | 41.8 | 47.9 | 57.7 |
| Age | 18-29 | 19.9 | 21.6 | 30.3 |
|  | 30-39 | 16.4 | 17.3 | 20.1 |
|  | 40-49 | 15.4 | 17.9 | 15.4 |
|  | 50-59 | 17.0 | 16.3 | 15.4 |
|  | 60-69 | 14.2 | 14.9 | 9.8 |
|  | 70+ | 17.0 | 11.9 | 9.0 |
| Education | Secondary school highest level | 34.2 | 28.3 | 29.0 |
|  | Further education | 65.8 | 71.7 | 71.0 |
| Marital status | Married/ de fecto | 58.7 | 56.4 | 48.5 |
|  | Single | 25.9 | 29.5 | 34.1 |
|  | Separated/Divorced | 9.6 | 9.7 | 11.4 |
|  | Widowed | 5.4 | 3.0 | 3.1 |
|  | Prefer not to say | 0.4 | 1.3 | 3.1 |
| Household income (AUD) | 0 to 80,000 | 72.0 | 73.0 | 69.7 |
|  | 80,001 + | 14.4 | 16.8 | 15.9 |
|  | Prefer not to say | 13.6 | 10.2 | 14.4 |
| State | Australian Capital Territory | 1.2 | 2.3 | 1.3 |
|  | New South Wales | 32.5 | 31.0 | 29.9 |
|  | Northern Territory | 0.8 | 0.6 | 1.7 |
|  | Queensland | 21.3 | 19.0 | 17.5 |
|  | South Australia | 11.0 | 7.5 | 3.8 |
|  | Tasmania | 2.2 | 2.7 | 4.7 |
|  | Victoria | 22.7 | 28.0 | 33.3 |
|  | Western Australia | 8.3 | 8.9 | 7.7 |

Note. Full = full attenders; Partial = partial attenders; None = Non-attender
